# The Role of Exercise in Cancer-Related Sarcopenia and Sarcopenic Obesity

**DOI:** 10.3390/cancers15245856

**Published:** 2023-12-15

**Authors:** Argyro Papadopetraki, Antonios Giannopoulos, Maria Maridaki, Flora Zagouri, Stavroula Droufakou, Michael Koutsilieris, Anastassios Philippou

**Affiliations:** 1 Department of Physiology, Medical School, National and Kapodistrian University of Athens, 115 27 Athens, Greece; argpapa@med.uoa.gr (A.P.);; 2 Section of Sports Medicine, Department of Community Medicine & Rehabilitation, Umeå University, 901 87 Umeå, Sweden; a.giannopoulos@lboro.ac.uk; 3 National Centre for Sport and Exercise Medicine (NCSEM), School of Sport, Exercise and Health Sciences, Loughborough University, Leicestershire LE11 3TU, UK; 4 Faculty of Physical Education and Sport Science, National and Kapodistrian University of Athens, 172 37 Dafne, Greece; 5 Department of Clinical Therapeutics, Alexandra Hospital, Medical School, National and Kapodistrian University of Athens, 115 28 Athens, Greece; 6 Medical Oncology Department, Iaso General Clinic, 151 23 Athens, Greece; s_droufakou@yahoo.com

**Keywords:** sarcopenia, sarcopenic obesity, cancer, exercise, physical activity, muscle atrophy, muscle wasting, weakness, body composition

## Abstract

**Simple Summary:**

Sarcopenia is a serious clinical condition experienced by many oncology patients as a disease and/or treatment-related adverse event that threatens their quality of life and survival. However, the assessment of body composition has not been incorporated into daily clinical practice and sarcopenia is either underdiagnosed or diagnosed at an advanced stage. Physical exercise offers significant benefits against sarcopenia, in terms of both prevention and treatment. In this review, the ways of assessing sarcopenia and sarcopenic obesity, their prognostic value, and their relation to the toxicity of the anticancer treatments are discussed. We also describe mechanisms by which exercise can counteract sarcopenia, and the potential role of myokines in the preservation of muscle mass.

**Abstract:**

One of the most common adverse effects of cancer and its therapeutic strategies is sarcopenia, a condition which is characterised by excess muscle wasting and muscle strength loss due to the disrupted muscle homeostasis. Moreover, cancer-related sarcopenia may be combined with the increased deposition of fat mass, a syndrome called cancer-associated sarcopenic obesity. Both clinical conditions have significant clinical importance and can predict disease progression and survival. A growing body of evidence supports the claim that physical exercise is a safe and effective complementary therapy for oncology patients which can limit the cancer- and its treatment-related muscle catabolism and promote the maintenance of muscle mass. Moreover, even after the onset of sarcopenia, exercise interventions can counterbalance the muscle mass loss and improve the clinical appearance and quality of life of cancer patients. The aim of this narrative review was to describe the various pathophysiological mechanisms, such as protein synthesis, mitochondrial function, inflammatory response, and the hypothalamic–pituitary–adrenal axis, which are regulated by exercise and contribute to the management of sarcopenia and sarcopenic obesity. Moreover, myokines, factors produced by and released from exercising muscles, are being discussed as they appear to play an important role in mediating the beneficial effects of exercise against sarcopenia.

## 1. Introduction

Epidemiological studies and basic research evidence have revealed the close relationship between inflammation and cancer pathogenesis. Indeed, inflammation has been identified as the seventh hallmark of cancer, and two distinct pathways have been described to elucidate the complex link between inflammation and cancer onset and evolution [1]. In the intrinsic pathway, genetic alterations initiate the formation of an inflammatory milieu, while in the extrinsic pathway, inflammation facilitates cancer development, progression and metastasis [2]. Moreover, a wide variety of pro-inflammatory factors, e.g., interleukin-6 (IL-6), interleukin-1 (IL-1), tumour necrosis factor-alpha (TNF-a), and transforming growth factor-β (TGF-β), which are secreted by cancer and tumour-associated immune cells, contribute to the occurrence of disease-associated adverse outcomes that deteriorate patients’ functional ability and quality of life [3,4]. These inflammatory-related side effects that the majority of cancer patients suffer from include fever, fatigue, haematological toxicity, malnutrition, weight loss, and increased adipose tissue deposition [5]. 

In addition, there is solid evidence that the undoubtable benefits of anticancer therapies against the evolution and recurrence of malignancies are also accompanied by a variety of side effects, which threaten treatment adherence and success [4]. For instance, chemotherapeutic drugs, such as alkylating agents, anti-metabolites, and anti-tumour antibiotics, which are widely used for cancer patients, can result in cardiotoxicity, nephrotoxicity, fatigue, cachexia, muscle wasting, leukopenia, neutropenia, anorexia, and gastrointestinal issues [6,7]. Furthermore, fibrosis, atrophy, and neural damage are considered common late effects of radiotherapy [8], while severe endocrine adverse effects are associated with anticancer hormonal therapies. Indeed, depending on the sex, age, and the mechanisms of action of hormonal therapy, patients may experience, among other symptoms, hot flashes, sexual dysfunction, depression, weight gain, bone density loss, and musculoskeletal symptoms [9,10,11]. Importantly, patients diagnosed with hormone-sensitive cancers may need to receive endocrine therapy for years and despite the fact that the adverse effects of hormone therapies are mild, compared to those of chemotherapies, they persist for long periods of time [12].

Sarcopenia, i.e., the loss of skeletal muscle mass and strength, often combined with the increased deposition of fat mass (sarcopenic obesity), is one of the most common cancer- and treatment-related side effects that oncology patients experience and its incidence ranges from 14% to 79%, while it depends on the age, sex, and type of cancer [13]. In particular, patients considered to be at the highest risk of developing sarcopenia are those with pancreatic and esophagogastric cancer, as sarcopenia is observed in up to 63% and 79% of these patients, respectively [14,15]. In breast cancer, which is the most common type of cancer among women worldwide, the incidence of sarcopenia occurrence is quite low (14–25.5%) [16,17], while in lung cancer, which has the highest incidence in men, the rates of sarcopenia for non-small-cell lung cancer and small-cell lung cancer are 43% and 52%, respectively [18].

Interestingly, a growing body of evidence supports the claim that physical exercise is a safe and effective complementary therapy for oncology patients, which can limit the cancer- and its treatment-related muscle catabolism and promote the maintenance of muscle mass. Moreover, even after the onset of sarcopenia, exercise interventions can counterbalance the muscle mass loss and improve the clinical appearance and quality of life of cancer patients. Thus, the aim of this narrative review was first to characterise the cancer-related sarcopenia and sarcopenic obesity, and describe the updated methods of their assessment, and subsequently discuss the implementation of exercise programs in sarcopenic cancer patients and the mechanisms by which exercise exerts beneficial effects against sarcopenia. For this purpose, the PubMed and Google Scholar databases were searched to collect the related studies, using the keywords sarcopenia, sarcopenic obesity, cancer, exercise, physical activity, muscle atrophy, muscle wasting, weakness, and body composition.

## 2. Sarcopenia

The term “sarcopenia” describes a syndrome characterised by the loss of skeletal muscle mass and strength and is derived from the Greek words “sarx” and “penia”, which mean “flesh” and “loss” [19]. In a recent consensus, definitions from various scientific groups and societies, the reduced functional ability, such as the low gait speed, and the increased fatigue, and the risk of falls have been included in the definition of sarcopenia [19,20,21,22,23]. It should be mentioned that sarcopenia is being considered a key feature of cachexia that many oncology patients experience, and the two terms should not be confused with each other. In particular, cachexia is a complex syndrome characterised by systemic inflammation and involuntary weight loss, irrespectively of whether it is originated from the loss of skeletal muscle mass or adipose tissue, and it cannot be fully counterbalanced by conventional nutritional supplements or pharmacological interventions [24,25]. 

**Figure 1:**
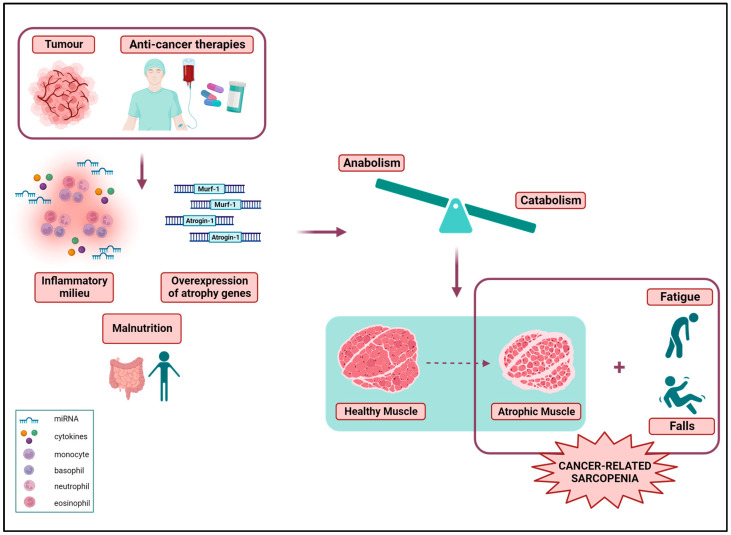
Sarcopenia is a serious clinical condition experienced by many oncology patients as a consequence of the disease and/or intensive anticancer therapies, which lead to an increased inflammatory milieu, the upregulation of muscle atrophy genes, and patients’ malnutrition. Catabolic processes exceed the anabolic ones, leading to muscle atrophy. Skeletal muscle mass loss combined with a reduced functional capacity, as reflected by fatigue and an increased risk of falls, constitute the cancer-associated sarcopenia. The figure was created with BioRender.com (accessed on 18 October 2023).

In the context of the pathophysiology of cancer-associated sarcopenia, skeletal muscle homeostasis is altered, and the balance between anabolism and catabolism, i.e., between protein synthesis and degradation, is disrupted, leading to progressive muscle wasting (Figure 1). Specifically, increased systemic levels of pro-inflammatory cytokines and micro-RNAs, and the overexpression of muscle atrophy genes mainly stimulate the ubiquitin–proteasome pathway (UPP) and the autophagy–lysosome system (ALS), activating several molecular pathways that ultimately lead to skeletal muscle atrophy [26,27,28]. As a result, the cross-sectional area (CSA) of skeletal muscle fibres decreases, especially in the type II fibres [29], while an intra- and inter-muscular infiltration of adipose tissue (myosteatosis) occurs. In addition, slow-twitch type I muscle fibres tend to switch to fast-twitch type II ones, shifting from an oxidative aerobic metabolism to a glycolytic anaerobic phenotype [30]. The muscle regeneration processes are also impaired due to the reduced number of the deranged properties of satellite cells [31]. Moreover, the metabolic activity of mitochondria is impaired, the mitophagy rate is increased, and the mitochondrial integrity is disrupted, leading to mitochondrial swelling [32,33]. 

Malnutrition is also a determinant in the pathogenesis of sarcopenia, which can occur as a consequence of both cancer and the anticancer treatments that result in difficulties in food consumption, impaired nutrient absorption, and increased episodes of diarrhoea or vomiting [34]. Malnutrition occurs in approximately 40% of oncology patients, while those with colorectal, gastrointestinal, lung, and head and neck cancer are considered the highest risk patients [35]. A recent meta-analysis revealed that the administration of nutritional supplements in patients with metastatic cancer experiencing malnutrition, sarcopenia, cachexia, and/or frailty could exert beneficial clinical outcomes, such as the modulation of inflammatory response, the promotion of muscle hypertrophy and the increase in muscle strength. Moreover, it is worth mentioning that superior effects are provoked by multimodal nutritional interventions, which combine the administration of various nutrients [36].

### The Assessment of Sarcopenia

In the absence of clear, specific, and widely accepted criteria for the evaluation and diagnosis of cancer-related sarcopenia, the assessment of skeletal muscle mass is considered an objective and major criterion for the identification of sarcopenia. A variety of methods are utilised to measure or estimate skeletal muscle mass among clinical populations, such as dual energy X-ray absorptiometry (DEXA), computed tomography (CT) scan, magnetic resonance imaging (MRI), ultrasonography (U) and bioelectrical impedance (BIA) [19,37,38]. Nevertheless, the heterogeneity of the methods used and the clinical populations in which skeletal muscle mass loss is assessed make it difficult to define uniform cut-off points for sarcopenia. 

CT scanning has been considered the gold standard method to assess skeletal muscle volume, possessing high reliability and repeatability [39]. However, it is not always the preferred method among clinical populations because of its cost and the exposure of the assessed individual to radiation. Nevertheless, these limitations may not apply to oncology patients, as most of them usually undergo CT scans as part of their disease diagnosis and follow-up [40]. To estimate whole-body skeletal muscle mass using CT scan, a cross-sectional CT image (cm^2^) is performed at the L3 level (third lumbar vertebra), as the abdominal muscle CSA is highly correlated with the total body muscle mass. This CSA divided by the square of the individual’s height represents the skeletal muscle index (SMI; cm^2^/m^2^) [37,40]. The most widely used cut-off values for diagnosing sarcopenia in oncology patients, based on SMI, are shown in Table 1. 

DEXA is also a common and precise method for estimating the lean body mass, with lower cost and substantially less ionising radiation than the CT scan [41]. Specifically, using a two-dimensional total body scan, the bone, fat, and lean tissue mass are separated [42]. The sum of muscle mass in upper and lower extremities is described as “appendicular skeletal muscle mass”, and when it is normalised with body height, it defines the appendicular skeletal muscle mass index (ASMI; kg/m^2^) [19]. Sex- and age-specific ASMI cut-off values for sarcopenia have been defined by Gould et al. and been accepted by the European Working Group on Sarcopenia in Older People (EWGSOP) [21,43]. Depending on the age of the clinical population assessed, sarcopenia thresholds range from 7.78 ± 0.88 kg/m^2^ to 8.67 ± 0.90 kg/m^2^ for men and from 6.45 ± 0.78 kg/m^2^ to 6.84 ± 0.80 kg/m^2^ for women [43] (Table 2). 

However, according to the definition of sarcopenia, measuring the skeletal muscle mass alone is not a complete approach for the assessment of sarcopenia and muscle strength should be also evaluated. Therefore, given the absence of cancer-specific guidelines regarding the optimal muscle strength evaluation tests, the recent consensus of EWGSOP should be utilised, which recommends universally accepted muscle strength assessments for the detection of a clinically significant loss of strength [21]. Indeed, handgrip-strength test is an inexpensive and easy-to-use tool accompanied by a well-established and validated usage protocol, that has been widely used in cancer patients [20,21]. Other assessing methods were also suggested in cases where the evaluation of handgrip strength is not feasible, or the assessment of lower extremities’ strength is preferred. For instance, the measurement of the isometric torque of lower limbs muscles using a dynamometer, the “chair-stand test” (which measures the time a patient needs to rise from a chair five consecutive times), or the “sit-to-stand test” (which measures how many times a patient can sit and stand up from a chair in 30 or 60 s) can be utilised [21]. Furthermore, according to the guidelines of the Asian Working Group for Sarcopenia (AWGS), the assessment of muscle strength (i.e., handgrip) and functional ability (i.e., chair-stand test) should precede the assessment of muscle mass [23]. Low muscle strength and functional ability is defined as a stage of “possible sarcopenia” and the individual should be referred for the further assessment of appendicular skeletal muscle mass [23]. Moreover, patients’ muscle strength values should be compared with age- and sex-specific normative values, or even with population-specific reference values if available, in order to identify the muscle weakness [44,45] (Table 3). According to the EWGSOP consensus, a clinically significant instance of dynapenia, a term used to describe muscle weakness, is defined when muscle strength values are below two standard deviations from the reference values [21,46]. 

**Table 1:** Skeletal muscle index (SMI) cut-off values to define cancer-related sarcopenia.

	Males	Females
Martin et al., 2013 [47]	<53 cm^2^/m^2^ (BMI ≥ 25 kg/m^2^)	<41 cm^2^/m^2^
	<43 cm^2^/m^2^ (BMI < 25 kg/m^2^)	
Prado et al., 2008 [48]	<52.4 cm^2^/m^2^	<38.5 cm^2^/m^2^

BMI: body mass index (kg/m^2^).

**Table 2:** Appendicular skeletal muscle mass index (ASMI) cut-off values to define cancer-related sarcopenia (modified from [43]).

Age (Years)	Males	Females
≥80	<7.78 ± 0.88 kg/m^2^	<6.45 ± 0.78 kg/m^2^
70–79	<8.22 ± 0.82 kg/m^2^	<6.60 ± 0.76 kg/m^2^
60–69	<8.53 ± 0.79 kg/m^2^	<6.66 ± 0.64 kg/m^2^
50–59	<8.77 ± 0.79 kg/m^2^	<6.84 ± 0.72 kg/m^2^
40–49	<8.96 ± 0.93 kg/m^2^	<6.82 ± 0.68 kg/m^2^
30–39	<8.92 ± 0.95 kg/m^2^	<6.83 ± 0.74 kg/m^2^
20–29	<8.67 ± 0.90 kg/m^2^	<6.84 ± 0.80 kg/m^2^

**Table 3:** Skeletal muscle strength and physical performance cut-off values to define cancer-related sarcopenia.

Handgrip Strength		
	Males	Females
Chen et al., 2020 [23]	<28 kg	<18 kg
Cruz-Jentoft et al., 2019 [21]	<27 kg	<16 kg
**Chair-Stand-Test**		
	Males	Females
Chen et al., 2020 [23]	≥12 s	≥12 s
**Gait Speed**		
	Males	Females
Cruz-Jentoft et al., 2019 [21]	≤0.8 m/s	≤0.8 m/s

## 3. Sarcopenic Obesity

Sarcopenic obesity (SO) is a unique clinical condition in which sarcopenia and obesity coexist. Thus, sarcopenic obese patients are characterised by the increased deposition of fat mass with the simultaneous loss of skeletal muscle mass and function [49,50]. The detection and diagnosis of sarcopenic obesity is a complicated procedure, for which there are not sufficient tools and cut-off criteria, especially in oncology patients. 

### The Assessment of Sarcopenic Obesity

Regarding obesity classification, most of the studies available today categorise patients according to the World Health Organization (WHO) classification, where those with a body mass index (BMI) ≥ 25 kg/m^2^ are considered overweight, and those with BMI ≥ 30 kg/m^2^ are considered obese [51]. Alternative threshold values have also been used (BMI ≥ 27.5 or 25 kg/m^2^) to define obese cancer patients [16,52,53]. However, regardless of which cut-off values are used to assess obesity, BMI does not take into account the body mass composition, and thus, the SO phenotype in cancer patients is often masked behind a normal or even overweight or obese BMI [54], highlighting the necessity of also assessing their body composition. When oncology patients lose weight as a result of either the disease or the therapeutic regimens, skeletal muscle loss is not expected to be equivalent to fat loss. Moreover, in some cases, simultaneously with the decrease in lean body mass, patients face a considerable weight gain [54,55]. 

In order to overcome incomplete and possibly ineffective diagnosis of SO, the European Society for Clinical Nutrition and Metabolism (ESPEN) and the European Association for the Study of Obesity (EASO) recently published a consensus statement for the definition of SO and its uniform diagnostic criteria [56]. According to this statement, people suspected or at high risk for SO should undergo a two-stage diagnostic procedure, i.e., screening and diagnosis. In the screening stage, patients should be monitored for a possible concurrence of obesity and indirect indicators of sarcopenia, such as impaired muscle function or repeated falls. For obesity assessment, BMI or waist circumference values should be compared to sex-, age-, and ethnicity-specific reference cut-off points. Subsequently, an alarming screening result should be followed by the diagnosis stage, in which, firstly, skeletal muscle strength should be assessed through widely used evaluation tests, such as the handgrip strength or the 30-s sit-to-stand test. In cases where reduced muscle strength is observed, body composition should be assessed via DEXA, CT scan, MRI, or BIA, with CT scan being preferred in oncology patients. When a sarcopenic obese phenotype is diagnosed, two-level staging may follow, depending on whether there are additional complications originated from the altered body’s composition. 

## 4. The Prognostic Value of Sarcopenia and Sarcopenic Obesity

A growing body of evidence supports that muscle wasting, the key component of sarcopenia and SO, should be regularly assessed among cancer patients, as it possesses great clinical significance and can predict disease progression and survival [57,58]. Despite the fact that body composition and body weight fluctuations of oncology patients are rarely assessed and thus sarcopenic and especially sarcopenic obese individuals are not detected on time.

In a recent study [59], the prognostic value of pre-treatment ASMI was examined in patients with advanced head and neck squamous cell carcinoma, who were under neoadjuvant or adjuvant chemoradiotherapy. The findings of this study revealed that the most extensive muscle mass loss was observed in the limbs, while ASMI was the only independent factor for the prognosis of a 2-year recurrence-free survival rate (RFSR) in these patients. In addition, when potential changes in skeletal muscle mass were investigated in patients receiving pre-operative chemoradiotherapy for rectal cancer [60], about 55% of them developed sarcopenia after the completion of chemoradiotherapy, while 25% of patients experienced severe muscle mass loss (>4.2%/100 days). Moreover, sarcopenia was associated with poor 5-year overall survival (sarcopenic patients: 72.5%, non-sarcopenic patients: 83.3%), while the subgroup who experienced the severe muscle mass loss exhibited the worst prognosis (65.2%).

In gastric cancer, which has one of the highest levels of sarcopenia incidence, SMI was shown to be a crucial predictor of overall survival for those patients who had undergone gastrectomy, while underweight patients with a BMI < 18.5 kg/m^2^ exhibited longer duration of hospitalisation [61]. Moreover, in pancreatic cancer patients, who are also at high risk of excessive muscle loss and impaired muscle function, sarcopenia and SO were associated with poor overall survival. Furthermore, sarcopenic obese patients suffered more from post-operative complications [62]. Interestingly, similar results concerning overall- and recurrence-free survival were also reported in even less advanced and in a lower risk type of cancers, such as a non-small-cell lung cancer [63,64] and breast cancer [16,65]. 

It is noteworthy to mention that sarcopenia does not occur exclusively in solid tumours but also in haematological malignancies and its early detection is of high importance. Indeed, in a single-centre retrospective analysis, it was revealed that 51% of adult patients with multiple myeloma before autologous haematopoietic cell transplantation (HCT) were defined as sarcopenic, while 23% were defined as sarcopenic obese. Interestingly, patients with sarcopenia, regardless of obesity status, had 12.5% more cardiac complications after autologous HCT [66]. In addition, when the SMI of 859 patients, who were scheduled to receive allogeneic HCT for leukaemia or myelodysplastic syndrome, was measured, it was unveiled that 33.7% of these patients had sarcopenia based on their SMI values. Furthermore, sarcopenia was found to be an independent risk factor leading to the 30% prevalence rate of 2-year non-relapse mortality, longer hospitalisation, and poorer overall survival [67]. 

## 5. Toxicity of Cancer Treatments in Sarcopenic and Sarcopenic Obese Patients

The total dose of the chemotherapeutic drugs administered is based on the anthropometric characteristics of each individual and is the product of the drug dosage/m^2^ multiplied by the body surface area (BSA), which is calculated using the equation: BSA = 0.007184 × Weight^0.425^ × Height^0.725^ [68]. However, it is well known that the same chemotherapeutic drugs and dosages may result in different side effects or different toxicity grades among oncology patients. Low skeletal muscle mass, which is a key component of sarcopenia, has been reported as an independent determinant of chemotherapy-related toxicity in patients with various types of malignancies, such as colorectal [69,70], breast [71], esophago-gastric [72], head and neck [73], or hepatic cancer [74]. The treatment-related adverse effects that have been associated with low muscle mass levels are haematological toxicity, gastrointestinal issues, neuropathies and prolonged hospitalisation [70,71]. Moreover, according to a recent meta-analysis, the reduced muscle mass is likely to lead to dose-limiting toxicities (DLT), i.e., the patients had to reduce or delay the drug dosage or even discontinue the specific treatment regimen [75]. 

The prevailing hypothesis regarding the mechanism by which low muscle mass increases the toxicity of chemotherapies is based on drug distribution throughout the human body. Specifically, according to the pharmacokinetic properties of many chemotherapeutic agents, especially the platinum-based ones, drugs are diffused through the fat-free compartments of the body, among which skeletal muscle tissue is the major distributor [73,76,77,78]. Hence, cancer patients with severely low muscle mass receive higher relative doses of chemotherapy drugs per kilogram of lean mass and cannot effectively diffuse the chemotherapeutic agents. This results in higher levels of chemo-substances in the circulation and, thus, in higher-treatment-related toxicities [16,78]. On the other hand, low muscle mass similarly does not influence other types of anticancer treatments, such as monoclonal antibodies, which are distributed by the blood and extracellular fluids due to their size and hydrophilic properties [79]. 

It is worth mentioning that, when low levels of lean body mass is combined with excessive fat mass deposition in sarcopenic obese cancer patients, the observed treatment toxicities are even worse compared to those observed in sarcopenic but not obese patients [54,71]. In addition, to the best of the authors’ knowledge, no study to date has compared the therapy-related toxicities in young sarcopenic versus elderly sarcopenic cancer patients. Nevertheless, there is sufficient indirect evidence supporting that older sarcopenic patients may experience more severe toxicities due to their comorbidities and the expected overall decline in the function of their physiological systems [80,81,82,83]. 

All the above observations, in combination with the fact that the dose of chemotherapeutic drugs is still determined based on BSA and not on body composition, might explain why patients with the same somatometric characteristics, i.e., the same BSA and BMI, experience different side effects and exhibit different grades of chemotherapeutic toxicities. Thus, it has been proposed that drug doses should be calculated based on skeletal muscle mass, in order to limit the treatment-related toxicities [84,85,86].

## 6. The Role of Exercise in Sarcopenia

In the last decade, a growing body of evidence has established that exercise is a safe and effective complementary therapy during cancer treatment, which limits cancer- and treatment-related side effects, thus helping the gradual incorporation of exercise in the daily routine of oncology patients [87,88,89,90,91]. The cancer guidelines recommend that cancer patients should start exercising as soon as possible, as the sooner they engage in exercise programs, the more the health-benefits they gain [92,93]. 

Sarcopenia is a common adverse effect that oncology patients experience due to muscle catabolism, which results from either the disease or the intensive anticancer therapies, and threatens their survival and overall prognosis [37]. Physical exercise has been proposed as the most effective non-pharmaceutic intervention for both the prevention and management of sarcopenia during the various stages of cancer progression [94,95]. Indeed, skeletal muscle mass is associated with physical activity levels and even only one week of bed rest can provoke significant skeletal muscle atrophy [96,97], while more pronounced skeletal muscle loss and functional ability impairment has been demonstrated after prolonged periods of physical inactivity [98,99]. 

**Figure 2:**
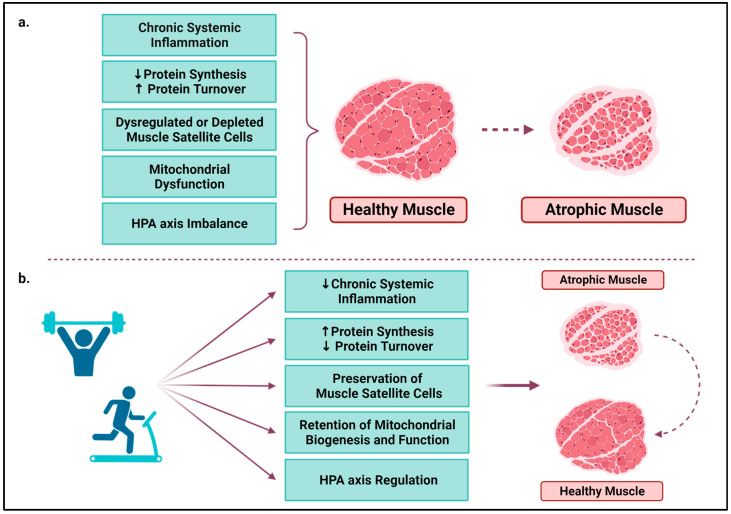
(**a**) Several mechanisms, directly or indirectly disturbed by the disease and cancer treatments, lead to cancer-related skeletal muscle wasting; (**b**) Physical exercise prevents muscle atrophy and/or reverses the sarcopenic phenotype of skeletal muscle by regulating these mechanisms, even in the context of cancer. HPA: hypothalamic–pituitary–adrenal axis; ↑: increase; ↓: decrease. The figure was created with BioRender.com (accessed on 18 October 2023).

The positive effects of exercise on the maintenance of muscle mass are due to its anabolic effects and the acute and/or chronic beneficial adaptations that causes in various physiological systems that can counterbalance the cancer-related catabolism (Table 4). More specifically, exercise regulates systemic chronic inflammation, protein synthesis, muscle stem cells and mitochondria function, as well as the hypothalamic–pituitary–adrenal (HPA) axis, which in turn, control skeletal muscle function even in the context of tumour development and progression (Figure 2). 

### 6.1. Chronic Systemic Inflammation

Chronic systemic inflammation creates a favourable environment for the onset and progression of several types of cancer [100], while it also triggers the development of cancer-related sarcopenia, through the actions of pro-inflammatory cytokines [101]. Indeed, cytokines such as IL-6, TNF-α, and TGF-β have been closely related to cancer-associated muscle wasting, since they disrupt muscle proteostasis, as well as mitochondrial biogenesis and function [102,103]. 

Interestingly, however, preclinical, and clinical evidence has linked exercise with lower levels of systemic inflammation in various types of cancer. This anti-inflammatory effect of exercise can be attributed to an acute inflammatory response of skeletal muscle, which in turn, leads to the production and secretion of anti-inflammatory factors [104]. Indeed, aerobic exercise has been shown to lower the levels of monocyte chemoattractant protein-1 (MCP-1), IL-6, and TNF-a in murine models with breast, colon, or Lewis lung carcinoma, and to mitigate muscle atrophy manifestations [105,106,107]. Moreover, resistance exercise in breast cancer patients undergoing treatment inhibited the increase in IL-6 and IL-6/interleukin-1 receptor antagonist (IL-1ra) ratio, which were associated with high levels of physical fatigue and pain in the control (non-exercising) group [108]. Similarly, a combination of resistance and high-intensity interval training in women with breast cancer undergoing chemotherapy was found to be more effective in decreasing plasma IL-6 levels, as compared with concurrent moderate-intensity aerobic and high-intensity interval training [109]. Moreover, it is noteworthy to mention that exercise intensity appears to have an important role in the regulation of cancer-related inflammation, as high-intensity exercise has been shown to lead to milder increases in C-reactive protein (CRP) and TNF-α in the plasma of breast cancer patients [110].

### 6.2. Protein Synthesis and Turnover

As previously mentioned, the imbalanced muscle homeostasis is a key hallmark of cancer-related sarcopenia, which leads to increased protein turnover [111]. However, preclinical evidence indicate that exercise training could regulate the cycle of protein synthesis and degradation even under the prism of cancer. Indeed, in colon-cancer-bearing mice, protein synthesis was promoted and CSA was preserved in response to aerobic exercise, through the activation of protein kinase B (Akt)/mammalian target of rapamycin (mTOR) signalling, a well-established molecular pathway that regulates protein turnover [112]. Moreover, in the same mouse cancer model, resistance exercise resulted in the increased mRNA expression of insulin-like growth factor-1 (IGF-1) Ea isoform and myogenin in mouse muscles [112], which are both implicated in skeletal muscle regeneration and hypertrophy processes [113,114,115,116,117]. Eccentric exercise also alleviated muscle atrophy manifestations in cachectic mice with intestinal neoplasia by triggering the mTOR pathway and mitigating the catabolic activity of AMP-activated protein kinase (AMPK) [118,119]. 

In addition, voluntary aerobic exercise (wheel running) in mice with colon cancer counteracted muscle wasting and prolonged their survival, via the downregulation of ubiquitin ligases Atrogin-1 and Murf-1 and the suppression of the autophagic degradation activity [120]. In another study, a similar 19-day protocol of voluntary wheel running resulted in the hypertrophy of type II muscle fibres in colon-cancer-bearing mice and led to the normalisation of paired box 7 (Pax7) protein expression, which dysregulation is related to the malfunction of muscle satellite cells (please see next paragraph) [121]. In terms of proteolytic pathways attenuation, the similar effects were observed after endurance training in lung-cancer-bearing mice receiving chemotherapy [107]. It is of high importance that the aforementioned preclinical evidence was confirmed in a 10-week clinical trial in female cancer patients, who are subjected to a combined exercise training complementary to chemotherapy [122].

### 6.3. Muscle Satellite Cells

Under normal conditions, muscle stem cells, which are localised between the sarcolemma and the basal lamina of myofibers, are activated and play a major role in the regeneration process [123]. On the oncology setting, the sarcolemma is damaged due to the increased circulation of inflammatory- and tumour-derived factors [31,124,125]. However, the regeneration process is compromised as satellite cells either become dysregulated and fail to proliferate and fuse properly due to the overexpression of the Pax7 gene [31], or are depleted due to the exposure to certain chemotherapeutic drugs such as doxorubicin [126]. 

Preliminary clinical findings in female patients with breast cancer undergoing chemotherapy support that exercise could preserve the number of muscle satellite cells per fibre [127]. The type of exercise is essential as the combination of resistance training with high-intensity interval training (HIIT) has emerged as the most beneficial for the preservation of satellite cells’ density, compared to moderate-intensity aerobic training combined with high-intensity interval training or the usual care [127]. These findings, accompanied by previous studies [128,129], suggest that exercise interventions in oncology patients that only consist of resistance training had no impact on the amount of muscle satellite cells.

### 6.4. Mitochondria

Dysregulated mitochondrial function and integrity are also implicated in the development of cancer-related sarcopenia as evidenced by murine models of cancer cachexia which displayed increased the mitochondrial emission of reactive oxygen species (ROS), fragmentation of mitochondrial network and poor respiratory function in their muscles [130]. Interestingly, all the aforementioned features implying mitochondrial degeneration appeared even before the onset of muscle wasting [130]. Clinical evidence in breast cancer patients undergoing chemotherapy confirmed that mitochondrial dysfunction occurs in their muscles [131], which is reflected in the reduced mitochondrial biogenesis, increased mitophagy, and altered cycles of fission and fusion [132]. Concerning the molecular mechanisms involved in cancer-associated mitochondrial disorganisation, in vitro studies have revealed that tumour-secreted factors alter their function through different mechanisms related to peroxisome proliferator-activated receptor gamma (PPAR-γ) signalling [133].

However, in mice with colon cancer that were subjected to moderate-intensity wheel-running for two weeks, mitochondrial biogenesis and activity were retained, as indicated by the upregulation of the peroxisome proliferator-activated receptor gamma coactivator 1-alpha (PGC-1a), as well as the increased content of the mitochondrial succinate dehydrogenase (SDH) and the regulator of cellular respiration cytochrome c [134]. Moreover, wheel running provoked an increased expression of mitochondrial complex II and IV in cancer-bearing mice, thus, sustaining mitochondria function [101]. Similarly, the beneficial effects of exercise on mitochondrial function of oncology patients are confirmed by human studies where different types of exercise resulted in enhanced citrate synthase activity [127] or the increased expression of a mitochondrial-derived peptide exerting exercise-mimetic activity, namely mitochondrial open reading frame of the 12S rRNA type-c (MOTS-c) [135]. 

### 6.5. HPA Axis

In addition to the direct action of inflammatory cytokines on muscle tissue, chronic systemic inflammation also indirectly affects muscle wasting through the dysregulation of the HPA axis under various clinical conditions, such as cancer. The HPA axis is the main regulator of glucocorticoids which are produced by the adrenal glands in response to adrenocorticotropic hormone (ACTH) secretion [136]. Glucocorticoids promote muscle mass loss and protein breakdown, while under normal conditions, their secretion is suppressed by negative feedback [137]. Thereby, the chronic activation of the HPA axis by pro-inflammatory interleukins such as IL-1 and IL-6 has been shown to exaggerate the major processes that mediate muscle wasting, namely the ubiquitin–proteasome pathway (UPP) and the autophagy–lysosome system (ALS) [138]. 

The regulation of the HPA axis is one of the mechanisms through which exercise can prevent or reverse extended muscle atrophy and sarcopenia due to cancer. Studies performed on different clinical populations have demonstrated that exercise could regulate the HPA axis response and attenuate its hyperactivity [139,140]. In fact, different types of aerobic exercise were shown to change the HPA axis-related markers in breast cancer survivors, such as cortisol or ACTH, in a similar manner to the alterations exhibited by healthy matched controls [141,142].

**Table 4:** Exercise and mechanisms counteracting cancer-related sarcopenia.

Author (Year)	Type of Research	Cancer Site	Sex	Type of Exercise	Exercise Characteristics	Intervention Period	Counteracting Mechanism
Mader et al. (2022) [105]	Preclinical	Breast	Female	Aerobic	Voluntary wheel running	4 weeks	↓Chronic systemic inflammation and ↑Mitochondrial biogenesis and function
Aoi et al. (2010) [106]	Preclinical	Colon	N/A	Aerobic	Treadmill; 3 times/week; 30 min; 20 m/min	6 weeks	↓Chronic systemic inflammation
Alves de Lima et al. (2020) [107]	Preclinical	Lewis lung	N/A	Aerobic	Treadmill; 5 times/week; 40–60 min; 60% maximum speed	2 or 3 weeks	↓Chronic systemic inflammation ↑Protein synthesis
Schmidt et al. (2016) [108]	Clinical	Breast	Female	Resistance exercise	2 times/week; 8 machine-based exercises; 3 sets × 8–12 reps; 60–80% of 1-RM	12 weeks	↓Chronic systemic inflammation
Hiensch et al. (2021) [109]	Clinical	Breast	Female	HIIT and resistance exercise	2 times/week; 8 resistance exercises (2–3 sets × 8–12 reps; 70–80% of 1-RM) followed by 3 × 3 min bouts of HIIT	16 weeks	↓Chronic systemic inflammation
Schauer et al. (2021) [110]	Clinical	Breast, prostate, or colorectal	Both	Group A: HIIT and resistance exercise; Group B: low-to-moderate aerobic and resistance exercise	Group A: 2 times/week; 2 min intervals at 80–90% HRR and 2 min active rest; 40–80 min weekly and 3 × 6 RM sets to 3 × 10 RM sets; Group B: 2 times/week; 10 min intervals at 40–50% HRR; 150 min weekly and 3 × 12 reps at 50% of 6 RM to 3 × 20 reps at 50% of 6 RM	6 months	↓Chronic systemic inflammation
Khamoui et al. (2016) [112]	Preclinical	Colon	Female	Group A: Aerobic; Group B: Resistance exercise	Group A: motorised wheels; 5 times/week; 60 min at 5–6.5 m/min; Group B: 3 times/week; 5 × 3 reps at 50% of body weight followed by 10% increases bi-weekly	11 weeks	↑Protein synthesis
Sato et al. (2019) [118]	Preclinical	Colorectal	Female	Eccentric (high-frequency electric stimulation)	10 sets × 6 reps, ~22 min	Acute or 2 weeks	↑Protein synthesis
Hardee et al. (2020) [119]	Preclinical	Colorectal	Male	eccentric (high-frequency electric stimulation)	10 sets × 6 reps every 48 h	2 weeks	↑Protein synthesis and ↑Mitochondrial Function
Pigna et al. (2016) [120]	Preclinical	Colon	Female	Aerobic	Voluntary wheel running	19 days	↑Protein synthesis
Coletti et al. (2016) [121]	Preclinical	Colon	Female	Aerobic	Voluntary wheel running	20 days	↑Protein synthesis and muscle satellite cells preservation
Møller et al. (2019) [122]	Clinical	Breast, head, and neck, rectal or sarcoma	Female	Aerobic and resistance exercise	3 times/week; 90 min; aerobic on ergometer bicycle and 6 resistance exercises	10 week	↑Protein synthesis and ↑Mitochondrial Function
Mijwel et al. (2018) [127]	Clinical	Breast	Female	HIIT and resistance exercise	2 times/week; 9 resistance exercises (2–3 sets × 8–12 reps; 70–80% of 1-RM) followed by 3 × 3 min bouts of HIIT (cycling) at 16–18 Borg scale	16 weeks	↑Muscle satellite cells and mitochondrial function
Ballarò et al. (2019) [134]	Preclinical	Colon	Both	Aerobic	3 days out of 4; 11 m/min for 45 min; wheel running	2 weeks	↑Protein synthesis and ↑Mitochondrial function
Dieli-Conwright et al. (2021) [135]	Clinical	Breast	Female	Aerobic and resistance exercise	3 times/week; aerobic at 65–80% HRmax; 30–50 min and 3 sets × 10 reps; 8 resistance exercises; 60% or 80% 1RM)	16 weeks	↑Mitochondrial function
Toohey et al. (2020) [141]	Clinical	Breast	Female	HIIT	3 times/week; 7 intervals × 30 s cycling (maximum effort) with 2 min active rest between intervals	12 weeks	HPA axis regulation
Evans et al. (2019) [142]	Clinical	Breast	Female	Aerobic	30 min cycling at 60% VO_2_peak	Acute	HPA axis regulation

Reps: repetitions; RM: repetition maximum; HIIT: high-intensity interval training; HRR: heart rate reserve; VO_2_peak: peak oxygen consumption; ↑: increase; ↓: decrease

## 7. Exercise Interventions after the Onset of Sarcopenia in Cancer Patients

As mentioned before, sarcopenia is a devastating health condition that a considerable amount of oncology patients will probably face at some point. Therefore, clinicians should advise their patients to engage in physical exercise programs to prevent the emergence of sarcopenia. However, “delayed” exercise interventions in patients who have already developed sarcopenia appears to be able to provide health benefits and improve their clinical appearance [46,143,144,145] (Table 5). Specifically, different types of supervised exercise were compared regarding their impact in sarcopenia features among sarcopenic obese patients undergoing adjuvant chemotherapy for breast cancer [46]. The results of this study documented that resistance exercise training for a total of 17 weeks had superior effects on muscle mass and strength compared with the usual care or aerobic exercise. Moreover, 26.2% of the patients reversed their sarcopenia status and consequently improved their self-reported quality of life, fatigue, and anaemia [46]. In addition, in another study, researchers investigated the effect of a 3-month resistance exercise intervention, with or without protein supplementation, in sarcopenia and quality of life of prostate cancer patients receiving androgen deviation therapy [143]. Resistance exercise attenuated sarcopenia for 28.3% of the patients, as evidenced by increased muscle strength and improved body composition and quality of life, while interestingly, protein supplementation had no positive effect [143]. 

Furthermore, similar manifestations were demonstrated after a combined exercise training intervention (both aerobic and resistance training), which was performed in sarcopenic obese breast cancer survivors, three times per week for a 4-month duration [145]. Indeed, significant improvements were noted in ASMI and obesity-related parameters, such as BMI, body weight, hip circumference, and body fat percentages, ameliorating the sarcopenic obese phenotype [145]. An intra-hospital, combined, exercise intervention was also carried out in which hepatoma patients with transcatheter arterial chemoembolisation participated in a low-to-moderate-intensity exercise training daily [144]. Even if the median exercise intensity was only 2.5 metabolic equivalents, it yielded significant clinical benefits in the intervention group, as SMI increased, Δcreatinine decreased and ΔeGFR (estimated glomerular filtration rate) elevated, indicating a reduced catabolism and improved kidney function [144]. 

The supervised exercise programs were well tolerated by cancer patients and the adherence ranged from 68.2% to 95%, highlighting their feasibility under the onset of sarcopenia. On the other hand, when a pre-operative unsupervised exercise intervention—combining both aerobic and resistance training with nutritional support—is applied in patients with gastric cancer, reduced compliance (50%) was reported [146]. However, given the small duration of the pre-rehabilitation program, great outcomes were reported in terms of muscle strength and the reversal of sarcopenia [146]. The analogous positive effect on muscle mass has been reported in a longer duration intervention involving aerobic exercise with no supervision, as in the pre-rehabilitation program in patients with colorectal cancer [147]. These findings are in contrast with the results of a previous study, regarding a 6-month unsupervised aerobic exercise training which was utilised as a complementary to anticancer treatment therapy and did not change the muscle CSA, only resulting in improvements in aerobic capacity and muscle strength [148]. 

Considering that sarcopenic oncology patients appear to be able to engage in exercise programs and that generally exercise under supervision is more beneficial for the health of cancer patients compared to the home-based unsupervised exercise training [87,149], they should be referred to supervised exercise in order to ensure that patients receive the maximum health benefits. In addition, several study results suggested that interventions employing resistance training are more effective in reversing sarcopenia [46,148,150]. However, it is better that oncology patients to exercise without supervision and stay active with any kind of exercise, rather than to remain inactive and refrain from all forms of exercise.

**Table 5:** Exercise Interventions after the onset of sarcopenia in cancer patients.

Author (Year) and Type of Research	Participants (=n)	Age and Sex	Type of Exercise	Intervention Period	Supervision	Nutrition Therapy	Main Outcomes
Adams et al. (2016) [46] randomised control trial	Breast cancer patients during chemotherapy (R.E.= 66, A.E. = 64, U.C= 70)	25.0–78.0 Females	R.E. (2 sets × 8–12 reps; 9 exercises, 60–70% 1RM)—A.E. (15 min 60% VO_2_peak and 45 min VO_2_peak)	3 times/week for a total of 9–24 weeks (median: 17 weeks)	Yes	No	R.E. reversed sarcopenia and dynapenia: ↑SMI, ↑Μuscle strength (upper and lower extremities), ↑QoL,↑fatigue, ↑anaemia
Dieli-Conwright et al. (2018) [145] randomised control trial	Breast cancer survivors (COMB. EX. = 50, U.C. = 50)	53 ± 10.4 Females	COMB. EX. (A.E.: 65–80% HRmax; 30–50 min and R.E.: 3 sets × 10 reps; 8 exercises; 60% or 80% 1RM)	3 times/week for a total of 16 weeks	Yes	No	COMB. EX. attenuated the sarcopenic obese phenotype: ↑ASMI, ↓ΒΜΙ, ↓Hip circumference, ↑Lean mass, ↓Fat mass, ↓Trunk fat
Dawson et al. (2018) [143] pilot randomised control trial	Prostate cancer patients on ADT (R.E. = 7, R.E and PRO = 6, PRO = 10, STR = 9)	67.5 ± 8.7 Males	R.E. (3 sets; 10 exercises) Weeks 1–2: 60% 1RM, 15 repetitions; weeks 3–4: 65–67% 1RM, 15–12 repetitions;weeks 5–6: 70% 1RM, 12–10 repetitions; weeks 7–8:75% 1RM, 10–8 repetitions; weeks 9–10: 80% 1RM, 10–8 repetitions; weeks 11–12: 83% 1RM, 8 repetitions	3 times/week for a total of 12 weeks	Yes	Yes	R.E improved sarcopenia prevalence: ↑Lean mass, ↓%body fat, ↑Muscle strength, ↑QoL; Protein supplementation did not offer additional benefits in improving body composition
Koya et al. (2019) [144] retrospective case–control study	Patients with hepatocellular carcinoma during chemoembolization period (COMB. EX. = 102, U.C. = 107)	60.0–91.0 Females/ males: 74/135	COMB. EX.: median 2.5 metabolic equivalents/20–40 min/day (R.E.: 3 sets × 10 reps; 60% 1RM or body weight and A.E.:10–15 min; 11–13 Borg scale and stretching and balance training)	Daily for 5–10 days (median: 7.5 days)	YES	YES	COMB. EX. improved clinical appearance: ↑SMI, ↓Δcreatinine, ↓ΔeGFR
Yamamoto et al. (2017) [146] pilot uncontrolled trial	Patients with gastric cancer during pre-operation period (COMB.EX. = 22)	75 ± 5 Females/ males: 12/10	COMB. EX.: handgrip(10 kg) 20 times/day; walking > 7500 steps/day; R.E.: 3 sets × 10 reps; 40–60% 1RM; 3 exercises	Daily for 7–26 days (median: 16 days)	NO	YES	COMB. EX.: ↑Handgrip, tendency to ↑SMI and ↑Gait speed
Moug et al. (2020) [147] randomised control trial	Patients with colorectal cancer during neoadjuvant chemoradiotherapy (EX. = 20, U.C = 24)	66.8 ± 9.6 Both sexes (64% males)	ΕΧ.: progressively increasing walking using pedometers	Daily for ≥13 weeks (median: 14 weeks)	NO	NO	EX improved sarcopenia-related parameters: EX.: 65% of patients ↑Muscle mass and 35% of patients ↓Muscle mass; U.C: 67% of patients ↓Muscle mass and 33% of patients ↑Muscle mass
Delrieu et al. (2021) [148] uncontrolled trial	Metastatic breast cancer survivors (EX. = 49)	54.9 ± 10.4 Females	ΕΧ.: progressively increasing walking using activity trackers	Daily for 6 months	NO	NO	EX improved physical performance but not body composition: ~CSA; ~lean mass; ~skeletal muscle radiodensity; ~SMI; ↑Aerobic capacity; ↑Muscle strength

EX: exercise; R.E.: resistance exercise; A.E.: aerobic exercise; COMB. EX.: combined exercise (aerobic and resistance training); U.C.: usual car; STR.: stretching; PRO.: protein supplementation; Reps: repetitions; RM: repetition maximum; HRmax: heart rate max; VO_2_peak: peak oxygen consumption; ADT: androgen deprivation therapy; ASMI: appendicular skeletal muscle mass index; SMI: skeletal muscle mass index; BMI: body mass index; QoL: quality of life; CSA: cross-sectional area; eGFR: estimated glomerular filtration rate; ↑: increase; ↓: decrease; ~: no change.

## 8. The Role of Myokines in the Reversal of Cancer-Related Sarcopenia

Skeletal muscle has been recognised as an endocrine tissue which interacts and communicated with other organs and tissues through the synthesis and secretion of bioactive molecules. The most prominent muscle-secreted bioactive molecules are the myokines, myomiRs, growth factors, chemokines, and exosomes, and muscle contraction enhances their production and secretion [151]. These muscle-secreted factors that constitute the muscle secretome, act in a paracrine, autocrine, or endocrine manner and mediate, directly or indirectly, some of the beneficial effects of exercise on cancer patients’ clinical outcomes. In the context of sarcopenia, where myokine signalling has been proposed to be altered [152], skeletal muscle contraction plays a determining role in protein synthesis stimulation and muscle mass growth promotion [153,154]. Indeed, the number of myokines shown to counteract muscle mass loss, not only in healthy populations but also under the prism of cancer, is steadily increasing. 

The promising role of a new myokine, fibrinogen C domain containing 1 (FIBCD1), in maintaining muscle mass in patients with cancer was very recently identified [155]. Specifically, FIBCD1 seems to preserves muscle wasting via ERK signalling, which has a very well described role in the promotion of muscle cell hypertrophy and the maintenance of neuromuscular junctions [156]. The exogenous administration of recombinant FIBCD1 in cachectic mice with Lewis lung carcinoma or melanoma, mitigated the cancer-related muscle atrophy, without promoting tumour growth and progression [155]. 

Nevertheless, the most widely studied myokine for its role in sarcopenia is myostatin (Mstn). Myostatin expression is muscle-specific and leads to protein breakdown and atrophy via binding to activin receptor type-IIB (ActRIIB), a TGF-β superfamily receptor [157,158]. Particularly, various types of exercise, but especially resistance training [159,160], suppress the expression of myostatin, which subsequently downregulates the atrophy genes Murf-1 and atrogin-1 [161]. Specifically concerning the cancer-related sarcopenia, high levels of myostatin have been associated with pronounced muscle wasting in several cancerous animal models such as melanoma, colon, or Lewis lung cancer [162,163,164]. However, the pharmacological blockade of myostatin, not only in animal models but also in human clinical trials, resulted in increased lean body mass, reduced fat mass, and improved functional ability without causing considerable adverse effects [165]. 

Moreover, activin A (ActA), a member of the TGF-β superfamily encoded by INHBA gene, has also been recognised as a myokine which is produced and released from skeletal myocytes in response to muscle contraction [166,167]. ActA retains similar actions with myostatin, since it utilises the ActRIIB receptor, and thus regulates the cancer-related muscle atrophy and cachexia [168]. Indeed, several studies have connected high levels of ActA with increased metastases, poorer overall or progression free survival, and low-muscle mass levels in oncology patients [168,169,170].

Furthermore, stromal derived factor 1 (SDF1) is encoded by the CXCL12 gene and its expression by skeletal muscles as well as its release into circulation is induced by exercise training [171,172,173]. The upregulation of SDF-1 enhances muscle fibre hypertrophy while being inversely correlated with the expression of Murf-1 and atrogin-1 in cancer patients’ muscles [174]. In addition, low levels of SDF-1 were observed in atrophying muscles of different animal models for hepatoma, rectal, and colon cancer [174]. 

Musclin, which was initially described as a bone-derived peptide, is a novel myokine triggered by aerobic exercise and encoded by the Osteocrin (Ostn) gene. Increasing evidence implicates musclin in cancer-related muscle atrophy, besides its prominent role in the promotion of aerobic capacity and mitochondrial biogenesis [175]. In line with the manifestations of other myokines, musclin’s concentration in the plasma of sarcopenic mice with renal carcinoma was very low and its expression was suppressed in their skeletal muscles. However, muscle wasting was preserved in tumour-bearing mice that were transfected with muslin-carrying plasmids [176]. 

It is worth mentioning that more myokines have been indirectly associated with cancer-related sarcopenia, as their role in muscle mass maintenance and muscle wasting attenuation is well established, and a growing body of evidence suggests that their expression is altered in cancer. For instance, myokines with tumour-suppressing features such as SPARC, Decorin, IL-6, and Irisin have been revealed that are also involved in the prevention of muscle atrophy and sarcopenia alleviation [177,178,179,180]. Overall, cancer patients should be referred to exercise interventions, as it is a promising complementary therapeutic approach, which countermeasures muscle wasting processes and protein catabolism, even after the onset of sarcopenia.

## 9. Conclusions and Future Perspectives

It is well established that sarcopenia is a clinical condition that many oncology patients experience either as a disease-associated outcome or as a side effect of anticancer therapies. Sarcopenic obesity is a subset of sarcopenia, in which patients display an obese phenotype along with all the distinct features of sarcopenia, such as reduced muscle mass and dynapenia. However, in the absence of cancer-specific criteria for sarcopenia, its diagnosis is based on the widely accepted criteria for assessing muscle mass and strength. Moreover, as there are no specific cut-off values for muscle mass or strength in cancer patients, the reference to the age-matched healthy population may lead to inaccurate conclusions and hyperdiagnosis. Thus, more careful studies, based on well-defined cohorts, are needed to establish norms specific to the type and site of cancer, eventually leading to a more objective assessment of oncology patients and making it easier for healthcare providers not to neglect the assessment of a possible sarcopenia status. The assessment and early detection of sarcopenia is of great importance as it associated with the hospitalisation period, the prognosis and survival of oncology patients, regardless of the type of malignancy and whether it is considered high-risk for the development of sarcopenia. Furthermore, sarcopenic or sarcopenic obese cancer patients sometimes experience decreased bone mineral density, a syndrome defined as osteosarcopenia, which has also been linked with impaired physical functioning and poor prognosis in oncology patients, especially in older adults [181,182,183].

Another very important element related to sarcopenia is body composition, a major component of patients’ physical fitness, which is associated with the toxicity levels of cancer treatments. Indeed, patients with lower muscle mass levels experience worse chemotherapy-related toxicities, as drug dosages are determined based on patients’ body weight and height without considering the proportions of their body mass and fat. Although further studies are needed to verify this aspect, based on the research evidence so far, it is strongly recommended that chemotherapeutic drug dosages are determined based on body composition rather than body surface area, as it might mitigate the chemotherapy-related toxicities. 

Exercise as a non-pharmaceutical complementary intervention plays a very important role in the prevention and/or reversal of cancer-related sarcopenia and is considered as the most promising intervention for its management, having no side effects. Indeed, numerous studies have revealed that appropriate exercise training programs could prevent the onset of sarcopenia in cancer patients by regulating key mechanisms that modulate muscle mass loss and weakness, such as HPA axis, systemic inflammation, protein synthesis, and mitochondrial function. Moreover, exercise training could also benefit cancer patients who are already sarcopenic by reducing the sarcopenia manifestations and improving their clinical appearance. Furthermore, myokines have a distinct role in cancer-related sarcopenia, as they mediate some of the beneficial effects of exercise associated with protein synthesis and turnover. 

Unfortunately, however, despite the strong research evidencing the potential of exercise training to counteract cancer- and its treatment-related adverse effects, most patients and cancer survivors are not regularly physically active, and results from studies with patients participating in exercise training programs are limited. Hence, there is an increasing demand for these patients to be referred by their clinicians for participation in personalised exercise programs. Moreover, further research, utilising targeted exercise intervention clinical trials and investigating the effectiveness of specific exercise programs, would make a decisive contribution to the necessary enrichment of scientific data regarding the relationship between optimum exercise dose and outcomes of sarcopenia and SO. 

Moreover, although many molecular pathways and intracellular interactions associated with muscle atrophy have been characterised, ethical considerations and clinical limitations for taking muscle biopsies from cancer patients with sarcopenia limit the available data for further elucidating the mechanisms mediating cancer-related development of sarcopenia in humans. Thus, further studies are needed to identify and characterise the molecular mechanisms underlying sarcopenia and SO in patients with cancer. This would contribute to the development of more effective interventions, including optimum physical exercise, for helping these patients to prevent and/or mitigate the disease- and its treatment-associated loss of muscle mass and function, eventually improving their physical function and quality of life.
